# Craniofacial growth and function in achondroplasia: a multimodal 3D study on 15 patients

**DOI:** 10.1186/s13023-023-02664-y

**Published:** 2023-04-18

**Authors:** Anne Morice, Maxime Taverne, Sophie Eché, Lucie Griffon, Brigitte Fauroux, Nicolas Leboulanger, Vincent Couloigner, Geneviève Baujat, Valérie Cormier-Daire, Arnaud Picard, Laurence Legeai-Mallet, Natacha Kadlub, Roman Hossein Khonsari

**Affiliations:** 1 Service de chirurgie maxillofaciale et chirurgie plastique, Centre de Référence Maladies Rares MAFACE, Faculté de Médecine, Hôpital Necker—Enfants Malades, Assistance Publique—Hôpitaux de Paris, Université Paris Cité, Paris, France; 2 Laboratoire ‘Forme et Croissance du Crâne’, Faculté de Médecine, Hôpital Necker—Enfants Malades, Assistance Publique—Hôpitaux de Paris, Université Paris Cité, Paris, France; 3 Molecular and Physiopathological Bases of Osteochondrodysplasia. INSERM UMR 1163, Imagine Institute, Paris, France; 4 Unité de ventilation non invasive et du sommeil de l’enfant, Faculté de Médecine, Hôpital Necker—Enfants Malades, Assistance Publique—Hôpitaux de Paris, Université Paris Cité, VIFASOM, Paris, EA France; 5 Service d’oto-rhino-laryngologie et chirurgie cervico-faciale, Faculté de Médecine, Hôpital Necker—Enfants Malades, Assistance Publique—Hôpitaux de Paris, Université Paris Cité, Paris, France; 6 Centre de Référence des Maladies Osseuses Constitutionnelles, Service de Médecine Génomique des Maladies Rares, Hôpital Universitaire Necker-Enfants Malades, Paris, France

**Keywords:** Achondroplasia, *FGFR3*, Sleep apnoea, Geometric morphometrics, Cephalometrics, Principal component analysis, Craniofacial growth

## Abstract

**Background:**

Achondroplasia is the most frequent FGFR3-related chondrodysplasia, leading to rhizomelic dwarfism, craniofacial anomalies, stenosis of the foramen magnum, and sleep apnea. Craniofacial growth and its correlation with obstructive sleep apnea syndrome has not been assessed in achondroplasia. In this study, we provide a multimodal analysis of craniofacial growth and anatomo-functional correlations between craniofacial features and the severity of obstructive sleep apnea syndrome.

**Methods:**

A multimodal study was performed based on a paediatric cohort of 15 achondroplasia patients (mean age, 7.8 ± 3.3 years), including clinical and sleep study data, 2D cephalometrics, and 3D geometric morphometry analyses, based on CT-scans (mean age at CT-scan: patients, 4.9 ± 4.9 years; controls, 3.7 ± 4.2 years).

**Results:**

Craniofacial phenotype was characterized by maxillo-zygomatic retrusion, deep nasal root, and prominent forehead. 2D cephalometric studies showed constant maxillo-mandibular retrusion, with excessive vertical dimensions of the lower third of the face, and modifications of cranial base angles. All patients with available CT-scan had premature fusion of skull base synchondroses. 3D morphometric analyses showed more severe craniofacial phenotypes associated with increasing patient age, predominantly regarding the midface—with increased maxillary retrusion in older patients—and the skull base—with closure of the spheno-occipital angle. At the mandibular level, both the corpus and ramus showed shape modifications with age, with shortened anteroposterior mandibular length, as well as ramus and condylar region lengths. We report a significant correlation between the severity of maxillo-mandibular retrusion and obstructive sleep apnea syndrome (p < 0.01).

**Conclusions:**

Our study shows more severe craniofacial phenotypes at older ages, with increased maxillomandibular retrusion, and demonstrates a significant anatomo-functional correlation between the severity of midface and mandible craniofacial features and obstructive sleep apnea syndrome.

**Supplementary Information:**

The online version contains supplementary material available at 10.1186/s13023-023-02664-y.

## Background

Achondroplasia (ACH, OMIM 100,800) is the most frequent form of chondrodysplasia, occurring with an incidence ranging from 1/30 000 to 1/10 000 [1, 2]. Clinical presentation is characterized by rhizomelic dwarfism and craniofacial anomalies, including macrocephaly, frontal bossing, midface retrusion, mandibular malformations, and cranio-vertebral junction anomalies [1–4].

Achondroplasia is due to activating mutations in the Fibroblast Growth Factor Receptor 3 gene (*FGFR3*), consisting in a glycine-to-arginine substitution in the transmembrane domain of the receptor (position 380) in more than 97% of cases [5, 6]. Activating *FGFR3* mutations lead to disorganisation of growth plate cartilage, premature fusion of the skull base synchondroses and impaired bone elongation [3, 7–10].

The exact effect of *FGFR3* activating mutations on craniofacial skeletal phenotype and growth in ACH is not well understood. A better characterization of skull bone anomalies using 3D renderings could be of use to describe the multiple craniofacial anomalies occurring in this condition. In clinical practice, describing skull growth and form in ACH is crucial to evaluate the functional consequences of midfacial retrusion, mandibular malformations, in order to establish evidence-based treatment plans. Obstructive and central sleep apnea are among the most alarming functional issues in ACH, affecting 60% of patients [11, 12], and have been related to a sagittal shortening of the cranial base and stenosis of the foramen magnum [13]. Obstructive sleep apnea (OSA) [14] in ACH is partially due to midfacial retrusion. Currently, the relationship between skeletal craniofacial shape and functional respiratory anomalies is not well understood. Deciphering the craniofacial morphology and its growth in patient with ACH could help assess the beneficial effects of promising medical treatments that are currently being developed to counteract the effects of activating *FGFR3* mutations [15, 16].

The aim of this study was to better characterise and quantify the skeletal craniofacial phenotype in a cohort of 15 ACH patients, using clinical evaluation, 2D cephalometrics and 3D geometric morphometrics. We also investigated the relationship between craniofacial shape and sleep study parameters, to understand whether craniofacial anomalies could be predictive of the severity of obstructive sleep apnea.

## Methods

### Patients

All ACH patients were initially managed in the National Reference Centres for Congenital Bone Diseases (Centre de Référence Maladies Rares MOC) and for Cleft and Maxillofacial Malformations (Centre de Référence Maladies Rares MAFACE), located within Necker hospital. This retrospective study included 15 ACH patients from 2017 to 2021 with confirmed *FGFR3* gain-of-function mutations. We analysed clinical and orthodontics evaluation and photographs, respiratory polygraphic (PG) results, lateral cephalograms, and craniofacial computed tomographic (CT) scans (n = 11), being both performed before any skeletal craniofacial procedure, including orthodontic treatments. Patients whose ages at PG and at cephalograms were not similar (tolerance of 15 months maximum, i.e. 20% of age difference), were excluded from the study.

**Fig. 1:**
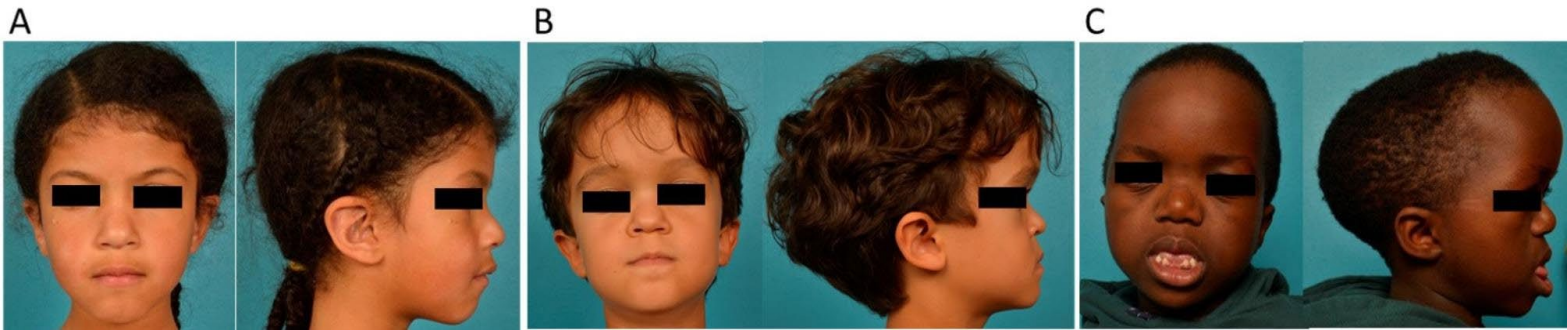
Clinical grading of morphological severity: maxillo-zygomatic retrusion: mild (patient A), moderate (patient B) and severe (patient C), normal nasal root (patient A), deep nasal root with moderate nasal bone hypoplasia (patient B), totally flattened nasal root with severe nasal bone hypoplasia (patient C), forehead: flattened (patient A), moderate convexity (patient B), markly prominent forehead (patient C)

To account for facial characteristics and to screen for potential clinical predictive factors of OSA, we used three morphological features—maxillo-zygomatic retrusion, deep nasal root, prominent forehead—with three grades of severity. Facial profile was sorted into three types (convex, concave, or flat) (Fig. 1). Occlusion was defined using the Angle classification [26].

### Controls

Controls (n = 11) were selected among age and gender-matched patients without any reported craniofacial anomalies. These patients underwent CT-scans performed within a short delay after benign trauma or infections that did not affect the studied regions.

### Cephalometric analysis

Cephalometric analysis was performed according to Delaire’s principles [17], using lateral cephalograms, with the software *DELAIRE CEPHALOMETRIE* (Blued’IS, Béthemont la Forêt, France). Fourteen landmarks were manually placed on each lateral cephalogram, defining 12 lines. Cranial and facial cephalometric analyses are described in supplementary data section (Figure S1 and Table S1).

### Investigation of skull and mandible shape

**Fig. 2:**
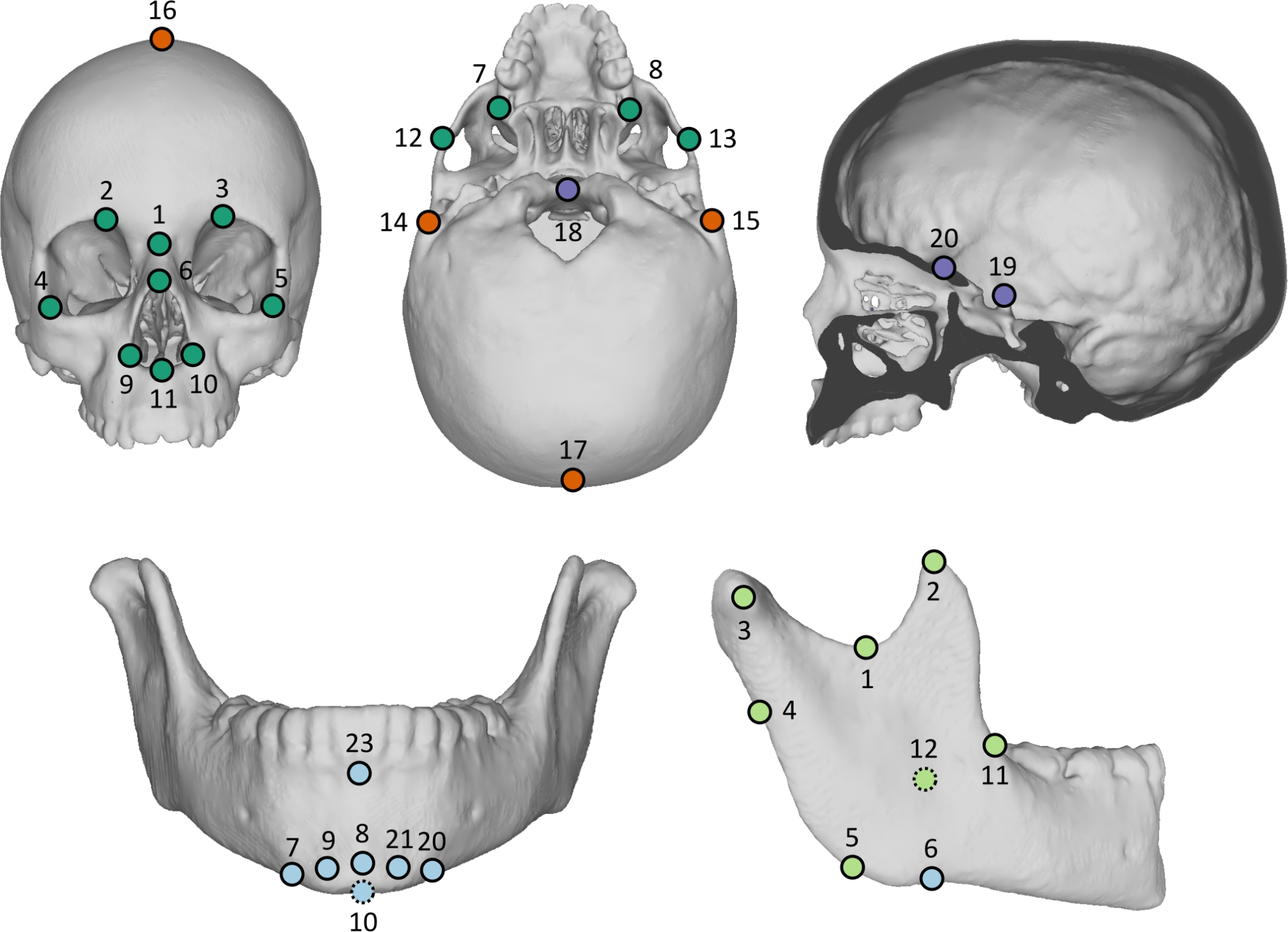
Anatomical landmarks placed on the skull (frontal and lower views; midline sagittal section) and mandible (frontal and lateral views). Landmarks 13–22 on the mandible are not visible, since they are symmetrically placed on the left side of the mandible. Color code for the skull: face = dark green; cranial vault = orange; skull base midline = purple. Color code for the mandible: mandibular corpus = blue; mandibular ramus = light green

An initial macroscopic analysis aimed at detecting potential premature fusion of skull vault sutures and skull base synchondroses, graded as follows: grade 1 (open), grade 2 (partially closed), and grade 3 (completely closed) [18–20]. Twenty landmarks were placed on the skull and twenty-three landmarks were placed on the mandible (Fig. 2, Table S2) using Avizo 2020 (Thermo Fisher Scientific, Waltham, MA, USA). Landmarks positioning was realized by the same expert, and preliminary study of intrarater reliability was performed to ensure repeatability of landmark annotation. Concretely, landmarks were placed 5 times on the skull and mandible of 3 patients. Three-dimensional coordinates were aligned and scaled by mean of a Procrustes superimposition, and Procrustes coordinates were compared among individuals using a multivariate analysis of variance with permutation procedure (1000 iterations). This enabled us to assess the relative importance of the variance due to intrarater bias and the variance existing between individuals. The tests resulted in clear differences between individuals (all p < 0.05), suggesting that intrarater bias was marginal. Landmarks supporting most of the intrarater variance were identified by displaying the thin-plate spline deformations along the axes of a Principal Component Analysis computed on the repeated landmarks of each individual. Special attention was further paid to these landmarks when the definitive annotation started. All computations and statistical analyses were performed using R [21]. Landmarks were aligned using Procrustes superimposition (*procSym*, *Morpho* package) [22], either with (1) standardisation of overall size (generating Procrustes coordinates), and (2) without scaling (generating Boas coordinates). With and without scaling, the 3D coordinates of the aligned points were combined into matrices to perform subsequent multivariate statistical analyses. All following analyses that generated theoretical 3D shapes used the mandible and the skull of a control individual as the reference shape. This reference shape was obtained by segmenting the CT-scan images of the individual with Avizo and by exporting the constructed volumes as 3D surface objects. Growth trajectories within each group of subjects were estimated from two-blocks partial least-squares regressions (2b-PLS), using *pls2B* from the *Morpho* package [22]. The first block corresponded to the Procrustes or Boas coordinates, and the second block corresponded to the log10-transformed age in years. Theoretical morphological variations along the statistically significant axis of covariation between shape and age were displayed using *tps3d* (*Morpho* package) [22]. Patterns of morphological changes during growth were compared between groups of individuals, first qualitatively, then quantitatively using the *compare.pls* (*geomorph* package) [23–25]. The covariation axes of the 2b-PLS regressions of Procrustes coordinates relative to age were used to extract theoretical morphologies at 6 different ages in each group: 0.5, 1, 3, 6, 9 and 12 years of age. Procrustes distances were computed for each landmark between the theoretical shapes in the two groups of patients, to estimate possible aggravation, defined as the increase in morphological differences between controls and patients with age. Deformation was then averaged within subsets of landmarks corresponding to 5 anatomical regions (Fig. 2). For the skull, three areas were considered: face (LM 1–13), cranial vault (LM 14–17), and skull base (LM 18–20). For the mandible, we considered two areas: mandibular ramus (LM 1–5, 11–19, 22) and mandibular corpus (the remaining landmarks). For each of these 5 anatomical regions, a logarithmic equation describing the evolution of deformation with age was generated. These growth equations enabled predicting deformation variations with age. The relationship between the intensity of morphological deformation and indices of apnea (see below, apnea-hypopnea index = AHI and obstructive AHI = OAHI) was investigated by computing stepwise regressions (using *stepAIC* from the *MASS* package) [26] between AHI or OAHI and the deformation of the five previously defined anatomical areas.

### Sleep parameters

Overnight PG with the recording of nasal flow, respiratory movements (bands), tracheal sound, body position, electrocardiogram, heart rate, pulse oximetry (SpO_2_), and transcutaneous carbon dioxide pressure (PtcCO2) were performed in room air (American Thoracic Society, 1996). Obstructive, central, mixed apnea and hypopnea were defined as previously described [11, 12]. The AHI was calculated as the sum of the apnea and hypopnea events per hour of total sleep. Sleep study was considered normal for AHI < 1.5 /hour [27]. PtcCO_2_ was recorded simultaneously by a Sentec monitor (Sentec, Therwill, Switzerland). The oxygen desaturation index (ODI) was considered abnormal when > 5 /hour. All available PG from all patients were considered to evaluate the evolution of sleep apnea during growth and after airways surgery.

### Relationships between cephalometric and sleep parameters

The correlation between each quantitative sleep parameter individually and all cephalometric variables was investigated through stepwise multiple regressions (using *step.AIC* from the *MASS* package. This enabled the best model to be retained by minimising the Akaike Information Criterion (AIC). Hence, the relative contributions of the explanatory variables to the variation of the dependent variable were assessed by computing the standardised (beta) coefficients.

Non-parametric three-way multivariate analyses of variance (MANOVAs with permutation procedure) enabled the estimation of the effects of the severity of maxillo-zygomatic retrusion, age and sex on the cephalometric and sleep parameters, using *procD.lm* from the *geomorph* package. Univariate pairwise permutation tests (alternative to the parametric univariate analyses of variance—ANOVA—and to *post-hoc* tests) were computed with *pairwise PermutationTest* from the *rcompanion* package [28]. These analyses aimed to investigate the effect of the factors that had shown significant influence in earlier multivariate analyses on individual cephalometric variables separately, and to perform pairwise mean comparison. The procedure applied a Bonferroni adjustment of the *p-value* to balance the biases of multiple comparisons.

## Results

### Patients

Fifteen ACH patients were included. Mean age at initial clinical evaluation was 7.8 ± 3.3 years; female / male ratio was 5/10. Genetic studies revealed the presence of a G380R mutation in *FGFR3* gene in all tested patients (n = 13). In the two remaining patients, born more than 15 years before the time of the study (i.e. 2021), molecular screening had not been performed, as genetic molecular confirmation was not mandatory at this period in these cases of typical clinical presentation of ACH. All patients presented a severe rhizomelic dwarfism, characterized by short limbs and trunk.

### Craniofacial morphological multimodal assessment

#### Clinical assessment

Four patients presented (i) mild, eight (ii) moderate and three (iii) severe maxillo-zygomatic retrusion (Fig. 1). Nasal root was deeply depressed in eleven patients and was less depressed in four patients. Forehead was flattened in 3/15, otherwise moderate convexity or marked prominent forehead affected 7/15 and 5/15 patients respectively. Profile was concave in 10/15, flat in 4/15, and convex in 1/15 patients.

#### Cephalometric analysis

Mean age at cephalometric analysis was 7.9 ± 3.2 years and was not statistically different from mean age at clinical evaluation. Maxillary retrusion (maxillary retroposition) and retrognathism (mandibular retroposition), in relation to cranio-adapted F1, affected all patients (n = 15). Skeletal Angle class was predominantly type III (n = 10/15), and less frequently I or II (n = 3 and 2/15, respectively). Gonial angle was mostly obtuse (n = 13) (relative to F3/F7 angle) and acute in 2/15 patients. All patients had excessive vertical dimension of the lower third of the face. Cranial base angles were abnormal in all patients, the anterior angle being obtuse (> 22°) in 8/15 patients, and the posterior angle acute (< 115°) in 13/15 (Table 1), accounting for modifications of the cranial base shape due to premature fusion of the skull base synchondroses (see below).

**Table 1:** Cephalometric analyses (n = 15 patients). SD: standard deviation. For the definition of the cephalometric parameters (C1, F1, F1M, F1m, C2, C4), see Fig. 1/ Suppl Table 1

		Mean ± SD	Definition
Age (years)		7.9 ± 3.2	
C1/F1 angle (degrees)		87.8 ± 5.3	
Maxillo-mandibular position	Maxillary position C1/f1M angle (relative to F1)	− 10.3 ± 4.9	maxillary retrusion (n = 15)
	Mandibular position (C1/f1m angle) (relative to F1)	− 8 ± 4.3	retrognathism (n = 15)
	Maxillo-mandibular discordance (f1M/1m angle)	− 2.2 ± 4.4	Angle class I/II/III (n = 3/2/10)
Gonial angle (degrees)		129.6 ± 20.32	open n = 13, closed n = 2 (relative to F3^F7 angle)
Vertical excess of the lower third of the face (%)		+ 6 ± 0.02	lower facial excess (n = 15)
Cranial base angles (degrees)	C1/C2 angle	23.8 ± 4.53 (20–22)	anterior angle of the cranial base (open n = 8, closed n = 5, normal n = 1)
	C1/C4 angle	111.2 ± 11.3 (115–120)	posterior angle of the cranial base (open n = 1, closed n = 13, normal n = 1)

### Craniofacial shape and growth: 3D-CT assessment

#### Cranial sutures and skull base synchondroses

Craniofacial CT-scans were available for 11/15 patients (female/male ratio: 3/8), with a mean age of 4.9 ± 4.9 years (range 0,2–13.6). Premature fusion of the squamo-sphenoidal suture affected 8/11 patients: either *in a partial* or *complete form* (4 patients each). A large anterior fontanelle was observed in 5/11 patients (all aged under 2 years), and 2/11 patients presented a mild fontanelle closure delay (ages 2.6 and 2.8 years).

All 11/15 patients presented with premature fusions (1) of the intra-sphenoidal synchondrosis (ISS) with 10/11 in a *complete* form (grade 3) and in 1/11 a *partial* form (grade 2); (2) of the spheno-occipital synchondrosis (SOS) with 9/11 in a *complete* (grade 3) and 2/11 in a *partial* form (grade 2), and (3) of the intra-occipital synchondrosis (IOS) bilaterally with 8/11 in a *complete* form (grade 3) and 3/11 in a *partial* form (grade 3). A *complete* fusion of the spheno-ethmoidal synchondrosis (grade 3) was observed in 7/11 patients, though it remained open (grade 1) in 4/11 patients.

#### Growth trajectories

**Fig. 3:**
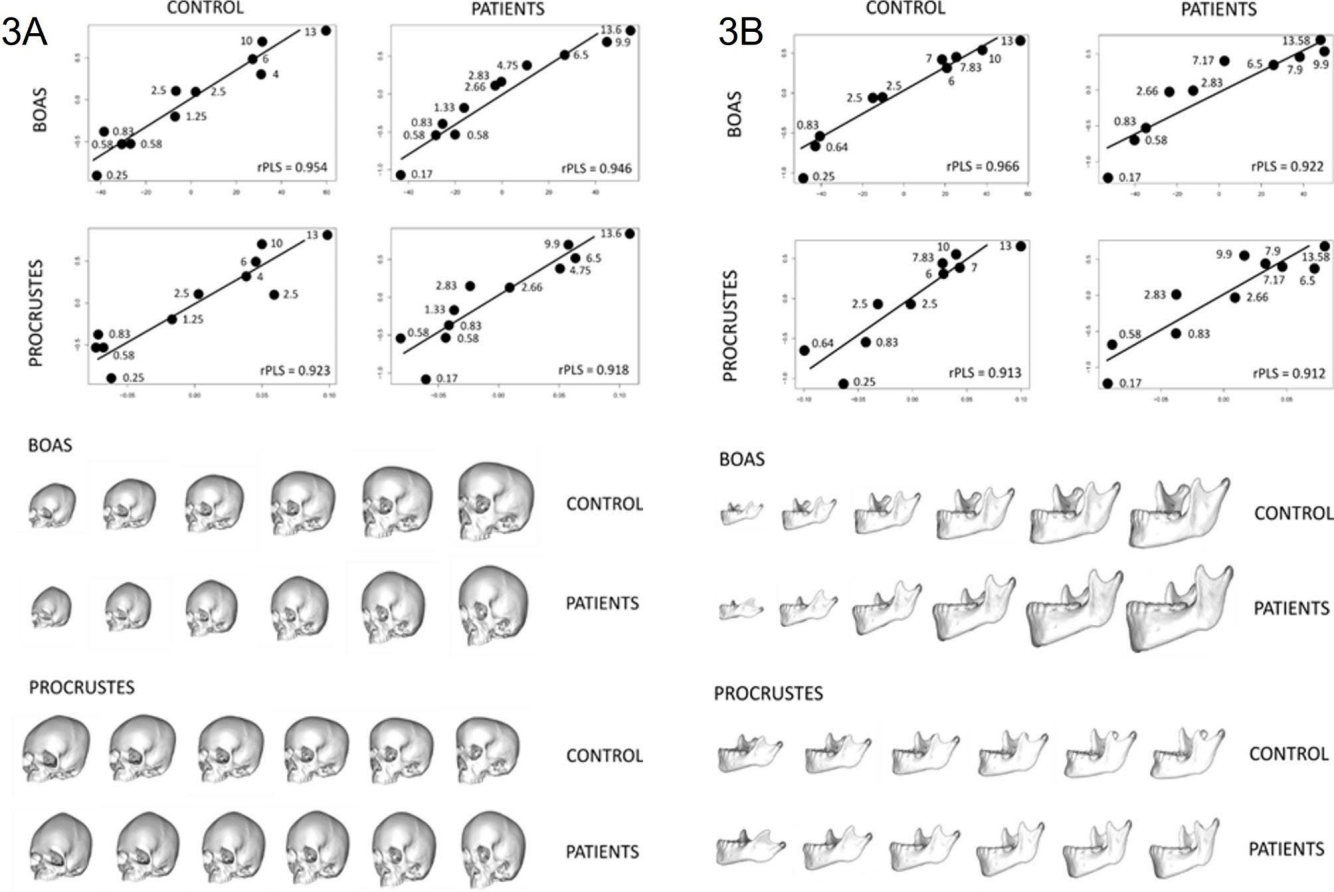
2b-PLS regressions between skull morphology and age (3 A), and between mandibular morphology and age (3B), describing growth trajectories in patients and controls, before (Boas coordinates) and after scaling (Procrustes coordinates). Numbers refer to age in years. Skull deformation series correspond to theoretical intermediate shapes along the covariation axis (negative values on the left side)

Both standardized (Procrustes coordinates) and non-standardized (Boas coordinates) skull and mandible shapes strongly covaried (rPLS > 0.9) with age within the ACH group and the control group (Table S3, Fig. 3). The strength of the covariation, provided by the rPLS index, was never significantly different between the two groups of patients, suggesting that intra-group variability in phenotype relative to age was comparable in the two cohorts.

The 2b-PLS regressions between Boas coordinates of the skull and age showed that, compared with controls, the growth of the skull in ACH was characterized by an overall retrusion of the midface and a forward tilting of the anterior aspect of the skull base, leading to a tightening of the space between skull base and the posterior part of the maxilla (Fig. 3).

More precisely, facial shape in ACH was characterised by a deep nasal root and a maxillo-zygomatic retrusion. The angulation of the skull base at the site of the SOS (with subsequent forward and downward tilting of the basisphenoid) and reduction of the skull base antero-posterior dimensions were associated with a shortening of the skull length. The orbits were vertically more elongated than in the control group. The skull vault was higher in the frontal region (which is mostly described by cephalometric landmark FPmid (Figs. 2 and 3 and Table S2), in older patients when compared with controls. Growth anomalies were also highlighted by 2b-PLS regressions between Procrustes skull coordinates and age, suggesting that these were not only due to size, but rather corresponded to disease-specific phenomena.

The 2b-PLS regressions between Boas coordinates of the mandible and age showed that mandible growth in ACH was characterized by a backwards shift of the symphysis and greater symphysis height (defined as the distance between cephalometric landmarks 8 Pog and InfDe, see Figs. 2 and 3 and Table S2). Additionally, in older patients when compared with younger ones, the mandibular ramus was narrower and more vertical overall, the notch of the sigmoid was more profound, the coronoid process and the condyle were more vertically positioned, and the segment between the retromolar region and the mandibular ramus was more concave. A decrease in the overall antero-posterior length of the mandible seemed to occur before teenage years, characterized by the shortening and verticalization of the condyle in older ACH patients. This decrease in length did not seem to occur in the first years of life. Similar results were obtained after scaling, suggesting that size moderately affected these morphological variations.

#### Phenotypic aggravation with age

**Fig. 4:**
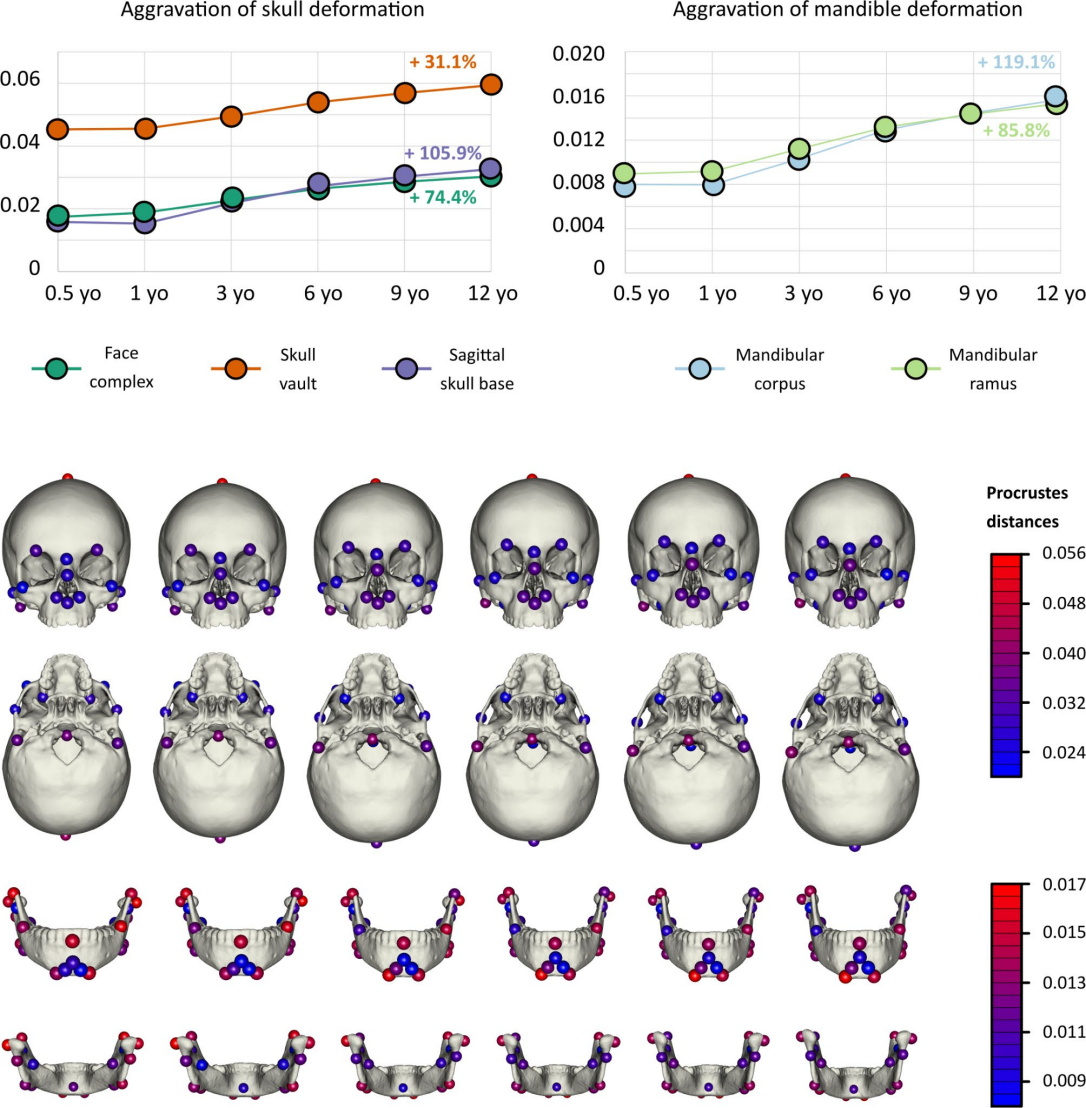
Patterns of phenotypic aggravation with age. Aggravation was defined as the increase of Procrustes distance for each landmark between controls and patients, by comparing the general growth trajectory of each group which was provided by the two-blocks partial least-squares regressions between Procrustes coordinates and age. Aggravation was averaged by anatomical region within both skull and mandible. In each graph, the x-axis was age in years (yo), and the y-axis was the Procrustes distance (no unit). The lower part of the figure represents theoretical morphological deformation of the skull (frontal and inferior views) and mandible (frontal and dorsal views) at 6 different ages (from 6 months to 12 years old), with landmarks highlighted in a gradient of color providing information about the intensity of the deformation in patients (Procrustes distance)

Skull vault showed the highest level of deformation between controls and patients (Fig. 4). Skull height increase was one of the main features of ACH. Nevertheless, the skull base and the face showed the most significant levels of aggravation with age (approximately + 75% to + 106% of phenotypic deformation from 6 months to 12 years old, respectively) (Table S4). The facial landmarks that showed the greatest aggravation in older patients when compared with younger ones were those surrounding the nostrils (InfExOL, InfNasApR, InfNasApL, InfNasMid, Fig. 2, Table S4).

Both the mandibular ramus and corpus showed comparable levels of phenotypic deformation between controls and patients. The most pronounced levels of aggravation were in the mandibular corpus (+ 119% of phenotypic deformation). When considering landmarks separately and not by anatomical region, it appeared that not all landmarks showed the same levels of aggravation with age. Overall, disparity in the levels of aggravation among landmarks was increased in older patients when compared with younger ones (Fig. 4). Within the mandibular ramus, the areas experiencing the greatest deformation were the coronoid process, the condyle and the gonion (CorR, CoExtR, GoR, CorL, CoExtL, GoL); and within the mandibular corpus, the most inferior part of the chin (MeR, MeL, Fig. 2 and Table S4).

No significant model was retained from the stepwise regressions between the intensity of shape deformation and indices of apnea (all p > 0.05).

### Functional assessment: obstructive sleep apnea syndrome

#### Polygraphic results and upper airway surgery

Mean age at sleep study was 7.8 ± 3.2 years, which was not statistically different from mean age at cephalometric study (difference 0,1491 ± 1,198). Sleep anomalies affected 80% of patients, with 33% patients having severe obstructive sleep apnea syndrome (AHI > = 10 events/hour) (Tables S5 and S6). Abnormal desaturations were observed in 13/15 patients. Apneas were mostly obstructive; the median index of central apnea was 0 (range 0–2.2). In our series, cranio-vertebral decompression had been performed in 4/15 patients with central apnea due to foramen magnum stenosis. In these cases, sleep analyses selected for the present study were performed after cranio-vertebral decompression.

**Fig. 5:**
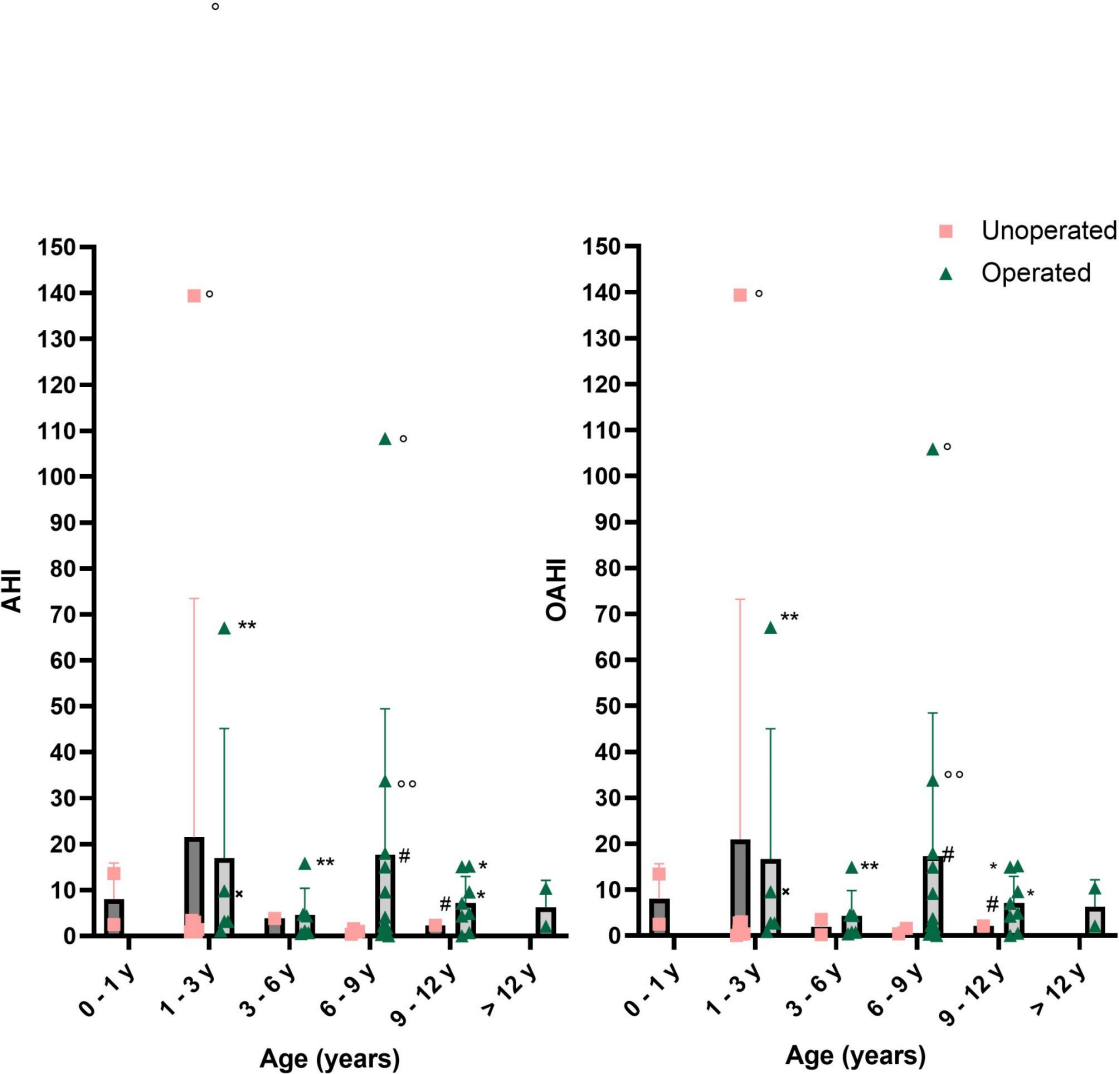
AHI and OAHI before and after upper airway surgery, per age groups. Values obtained from patients who required non-invasive CPAP ventilation at the time of the sleep study are labeled (a unique symbol is used for each patient). AHI: apnea hypopnea index, OAHI: obstructive apnea hypopnea index

Fourteen out of 15 patients benefited from upper airways surgery, mostly adeno-tonsillectomy and turbinectomy (Table S5). One out of 15 patients with severe ventilation disorders of multiple origins (pulmonary hypoplasia, obstructive apnea and central apnea due to upper spinal cord compression) had a tracheostomy and a cranio-vertebral decompression at the age of one and was decannulated two weeks post-operatively. Six out of 15 patients benefited from non-invasive continuous positive airway pressure (CPAP) ventilation, starting at the mean age of 4.9 ± 3.2 years, and CPAP had been stopped following a normal sleep study without CPAP in 3/6 of them (mean age 9 ± 1.7 years). In average, higher values of AHI and OAHI were observed at the ages 1–3 (19.5 ± 42.1, 13.5 ± 28.9 evens/hour, respectively) and 6–9 years of age (19.1 ± 42.2, 15.2 ± 30.3 events/hour, respectively) than at other ages (Fig. 5), although not significantly (p = 0.9, Kruskal-Wallis’s test).

#### Craniofacial phenotype and sleep disorders: anatomo-functional correlation

Stepwise regressions between each sleep parameter and the set of cephalometric variables retained three statistically significant models (Table S7). Greater AHI and OAHI values were both associated with more pronounced maxillary retrusion and retrognathism, and with smaller C1-C2 values. When maxillary and mandibular retrusion increased, SpO2 min decreased. Non-parametric MANOVAs with permutation detected no relationship between sleep study parameters and the severity of maxillo-zygomatic retrusion, sex, and age (all p > 0.05, Table S8). Cephalometric parameters were influenced by the grade of maxillo-zygomatic retrusion (p = 0.034; R² = 0.157; F = 2.538; Z = 1.904) and sex (p = 0.011; R² = 0.178; F = 2.874; Z = 2.287). More specifically, the univariate pairwise permutation tests did not reveal any significant relationship between the severity of maxillo-zygomatic retrusion and cephalometric parameters considered separately (Table S9). However, levels of maxillary retrusion and values of C1-C4 angles differed between sexes, with boys presenting with more severe maxillary retrusion and lower C1-C4 angle values than girls.

## Discussion

*FGFR3* is involved in craniofacial membranous and endochondral ossification processes [3, 29]. Gain-of-function *FGFR3* mutations lead to dwarfism (ACH, hypochondroplasia, and thanatophoric dysplasia) [5, 30, 31] but also craniofacial suture fusions (craniosynostoses: Muenke syndrome and Crouzon syndrome with *acanthosis nigricans*) [32, 33].

Even though all the ACH patients reported here presented similar craniofacial features (frontal bossing, macrocephaly, maxillary retrusion, deep nasal root, and prognathism) [3, 4, 34], we observed three grades of facial phenotype severity – ‘mild’, ‘moderate’ or ‘severe’ –, suggesting a phenotypic disparity in a genetic disease due in > 95% to a same G380R *FGFR3* mutation. All patients presented maxilla and mandible retrusion, an opening of gonial angle, a closure of the posterior skull base angles and a vertically elongated chin, confirming previous findings in ACH [34, 35].

Our 3D morphometric analyses suggested an aggravation of the craniofacial phenotype with age, even though our study is not longitudinal. The most affected craniofacial region was the midface, characterised by an increased maxillary retrusion and a deeper nasal root in older patients when compared with younger ones. Aggravation of midface retrusion was most probably related to the premature fusion of skull base synchondroses secondary to activating *FGFR3* mutations that impair cartilage homeostasis [3, 36], as observed in ACH mouse models. Normal synchondrosis fusion in humans follows a specific age-related sequence: ISS before the age of 2, IOS before the age of 7, spheno-ethmoidal synchondrosis before the age of 9, and SOS before puberty [20, 37, 38]. Premature fusion of skull base synchondroses was always observed in our series, at the site of ISS, SOS and IOS. Gradual premature fusion of skull base synchondroses contributes to anteroposterior facial growth restriction and subsequent maxillary and midfacial retrusion [19, 20, 39]. In our series, we observed abnormal angulations at the site of the SOS. These skull angle modifications may be related to the premature fusion of the skull base synchondroses, but we cannot exclude the influence of intrinsic brain anomalies, especially of the temporal region, as already reported in *FGFR* mutations [40, 41]. In addition, shape changes of the foramen magnum may also be involved in skull base anomalies, with secondary repercussions on the midface [3, 42].

Premature fusion of skull vault sutures was observed in 80% of the patients at the squamo-sphenoidal suture, and all patients under the age of 2 had a large anterior fontanelle, indicating potential anomalies in the membranous ossification of the skull vault [3]. In addition, brain anomalies and potential increased intracranial pressure may also worsen skull shape deformations and impair fontanelle closure and frontal bone ossification. However, previous ex vivo studies conducted on a mouse model of ACH, *Fgfr3*^*Y367Y/+*^, showed that the ossification delay of skull vault occurred independently of brain and cranial base parameters, suggesting an intrinsic influence of *FGFR3* gain-of-function mutations on membranous ossification [3]. Premature synostosis of one cranial suture constrains cranial growth at the site of the suture, and continuous growth of the underlying brain induces compensatory skull vault growth at the site of other non-fused cranial sutures, leading to skull deformations. Both the premature fusion of cranial sutures and ossification delays of frontal bones may play a role in the prominent forehead observed in ACH patients. In addition, this excessive frontal convexity is also accentuated by the presence of a nasal root depression at the nasofrontal junction, associated with the restricted anteroposterior growth of the skull base.

Obstructive sleep apnea in ACH can be related to multiple anomalies: volume reduction of the upper respiratory tract and nasopharyngeal stenosis (choanal stenosis, adenoids, and tonsils hypertrophy, as well as macroglossia), and airway muscles hypotonia [11, 12, 14, 35]. Here we report two main age periods associated with higher values of AHI and OAHI, i.e. 1–3 and 6–9 years, corresponding to the physiological higher incidence of adenoid and tonsils hypertrophy, respectively. Although an influence of nasopharyngeal obstructive factors in persisting obstructive sleep apnea has been reported in ACH, the surgical correction of these anomalies is often insufficient to treat apnea [11, 43]. This is possibly because bony anomalies including short skull base and midface retrusion persist. However, a correlation between craniofacial skeletal shape modifications and severity of OSA had never been confirmed in children with ACH. Here, we report significant correlations between maxillo-mandibular anomalies and AHI, OAHI, and hypoxia: greater AHI and OAHI and lower SpO_2_ min values were both associated with severe maxillary retrusion and retrognathism. In addition, higher AHI and OAHI significantly correlated with smaller C1-C2 angle values, highlighting correlation between skull base changes, maxillo-mandibular retrusion, and severity of obstructive sleep apnea. However, we cannot exclude structural and functional upper respiratory tract anomalies in ACH. Although it has been shown that *FGFR2* activating mutations lead to abnormal tracheal formation and segmentation [44–46], there is no data available documenting the impact of *FGFR3* activating mutations on respiratory tract formation. Therefore, a potential intrinsic impact of *FGFR3* mutations on airway formation, development, and function remains to be elucidated.

In addition to premature fusion of skull base synchondroses leading to anteroposterior craniofacial growth limitation, reduction of the nasopharyngeal airway flow itself also contributes to impair transverse and sagittal facial growth, as observed in mouth-breather non-syndromic children presenting chronic nasal obstruction [47]. In this context, functional defects due to *FGFR*-related anatomical anomalies most probably add to the ongoing effects of the *FGFR3* activating mutation in the aggravation of the phenotype with age.

The presence of an anatomo-functional correlation between maxillo-mandibular retrusion and OSA in ACH objectively stresses the clinical need for a specialized multidisciplinary follow-up in this condition with systematic craniomaxillofacial and orthodontic evaluations. Although the benefit of maxillary expansion in releasing nasal obstruction remains unclear, this orthodontic treatment is often recommended to treat palatal transversal insufficiency in ACH after the age of 6. Maxillary advancement is sometimes required, using either controversial orthodontic appliances (Delaire facemasks [48]) in the less severe cases, or surgery, in the cases of severe maxillary retrusion or functional symptoms (OSA, snoring). CPAP face masks may limit the feasibility of these treatments because of the external forces applied on the midface during maxillary advancement. Development of new CPAP appliances minimizing pressure on the midface should thus be considered in ACH patients [49].

Two patients benefited from maxillary expansion and/or maxillary protraction appliance (Delaire facemasks [8]). One of these patients underwent an interceptive Le Fort I osteotomy with distraction at the age of 10. All these procedures had been performed after 2D and 3D analyses used in the present study. At the time of the study (i.e. 2021), orthodontic treatment was also planned for five patients, and interceptive Le Fort I osteotomy with distraction was considered for two patients. Systematic re-assessment during growth was decided otherwise. Although our sample did not allow us to address this question, future studies should investigate the impact of orthodontic treatments and orthognathic surgery on OSA.

The limitations of our study are due to the small cohort with available cephalometric analyses, CT-scans, and sleep studies performed within the same short period of time to allow correlation studies. A longitudinal follow-up during growth, at different timepoints, will be necessary to confirm craniofacial phenotype modifications with age, and its correlations with the evolution of sleep disorders.

More generally, larger multicentric and prospective cohorts will be useful in future to understand whether and how additional potential factors (such as upper airway hypotonia or collapse, and macroglossia) could contribute to OSA in ACH.

## Conclusions

This study highlighted that achondroplasia leads to different degrees of craniofacial morphological and functional severity. We showed for the first time that more severe craniofacial phenotypes occur in older patients than in younger ones, which suggests an aggravation of craniofacial phenotypes during growth. We furthermore demonstrated an anatomo-functional correlation between the severity of maxillo-mandibular retrusion and OSA.

*FGFR*-related conditions due to activating mutations may soon benefit from medical treatments that will hopefully reduce the need for invasive surgical procedures. In this context, precise knowledge on the natural history of these conditions including ACH is crucial for adapting future treatment and assessing their efficiency, especially in resolving functional anomalies like OSA.

## Electronic supplementary material

Below is the link to the electronic supplementary material.

Supplementary Material 1

Supplementary Material 2

Supplementary Material 3

Supplementary Material 4

Supplementary Material 5

Supplementary Material 6

Supplementary Material 7

Supplementary Material 8

Supplementary Material 9

Supplementary Material 10

## Data Availability

All data generated or analysed during this study are included in this published article (and its supplementary information files).

## Abbreviations

ACH: achondroplasia
CPAP: Continuous Positive Airway Pressure
FGFR: Fibroblast Growth
OSA: obstructive sleep apnea
AHI: Apnea Hypopnea Index
OAHI: Obstructive Apnea Hypopnea Index
SpO_2_: pulse oximetry
PtcCO2: transcutaneous carbon dioxide pressure
ISS: intra-sphenoidal synchondrosis
SOS: spheno-occipital synchondrosis
