# Supplementary figures and images for: Craniofacial growth and function in achondroplasia: a multimodal 3D study on 15 patients
Source: Orphanet J Rare Dis. 2023 Apr 18;18:88. doi: 10.1186/s13023-023-02664-y (PMC10114380; doi:10.1186/s13023-023-02664-y)

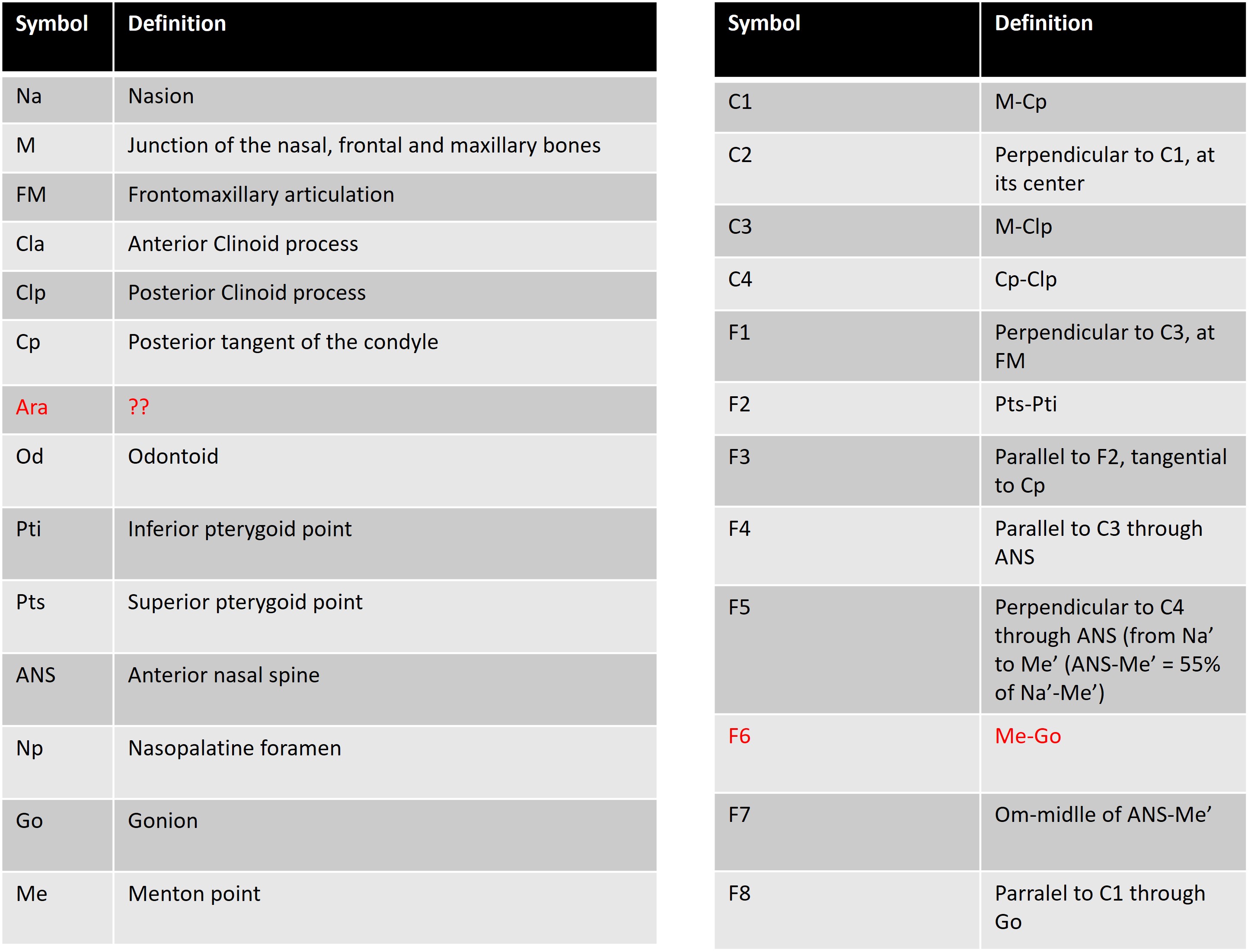

Supplement: Supplementary file 1 — Supplementary Material 1 [file 13023_2023_2664_MOESM1_ESM.jpg]

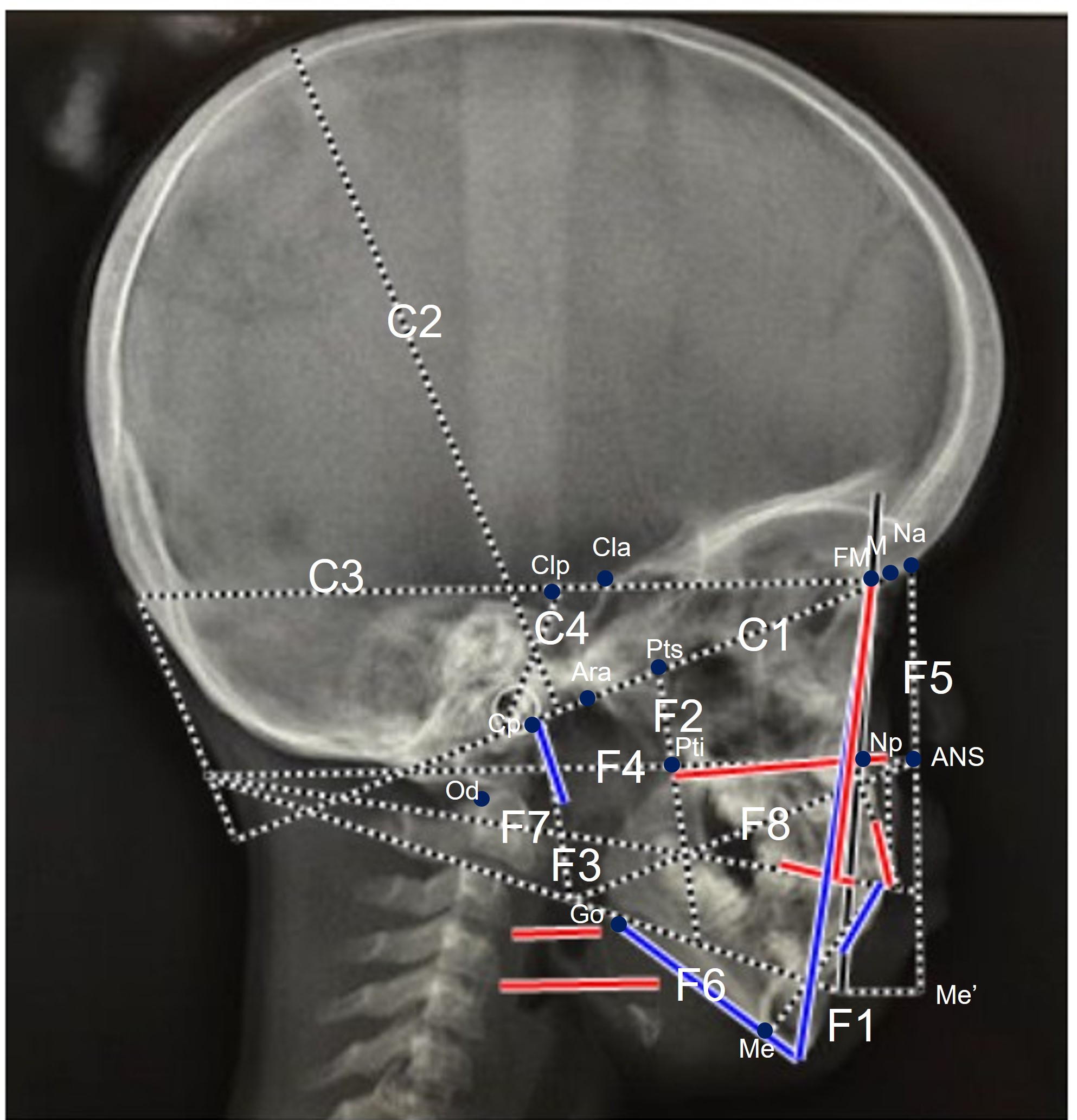

Supplement: Supplementary file 2 — Supplementary Material 2 [file 13023_2023_2664_MOESM2_ESM.jpg]

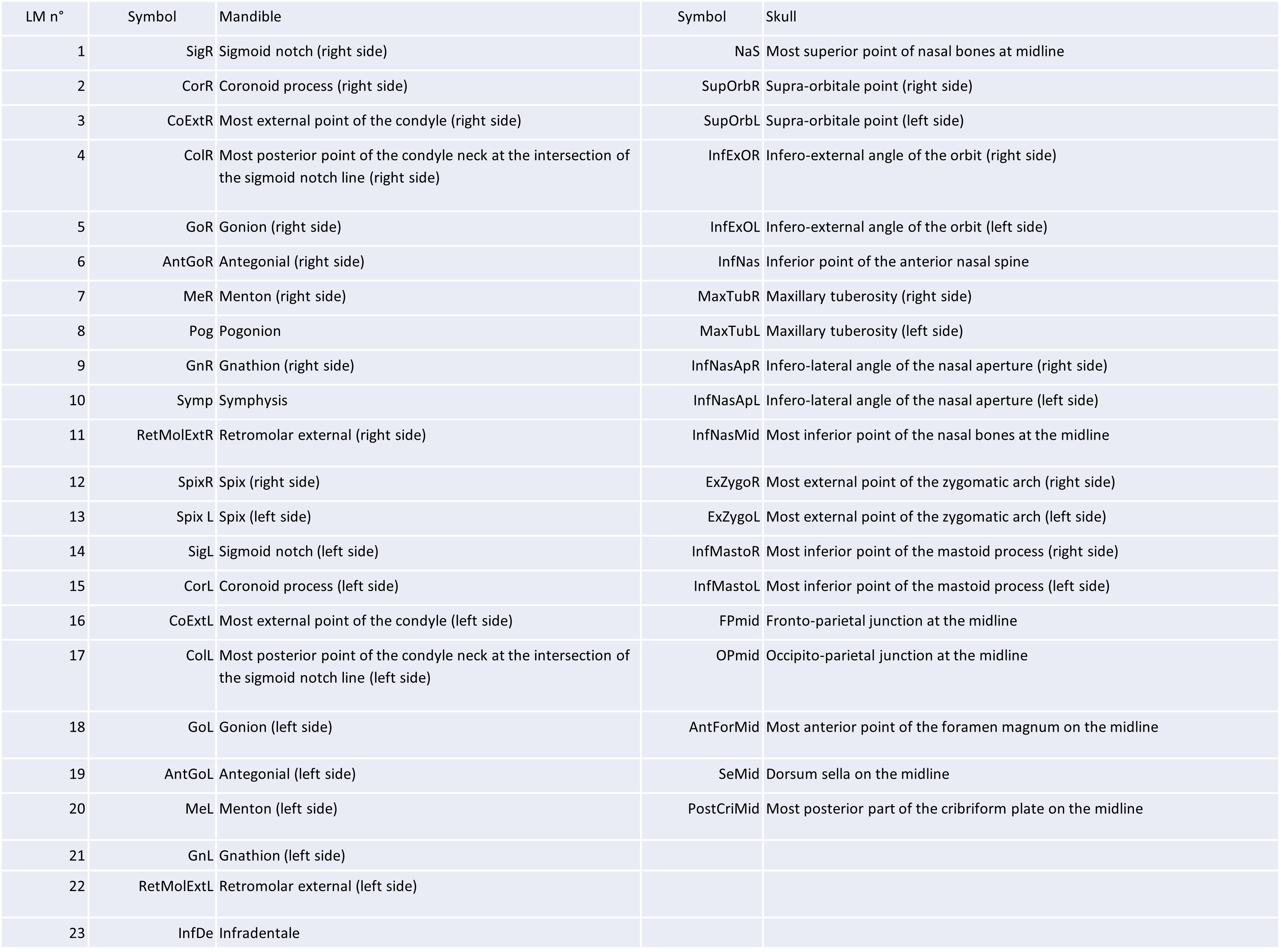

Supplement: Supplementary file 3 — Supplementary Material 3 [file 13023_2023_2664_MOESM3_ESM.jpg]

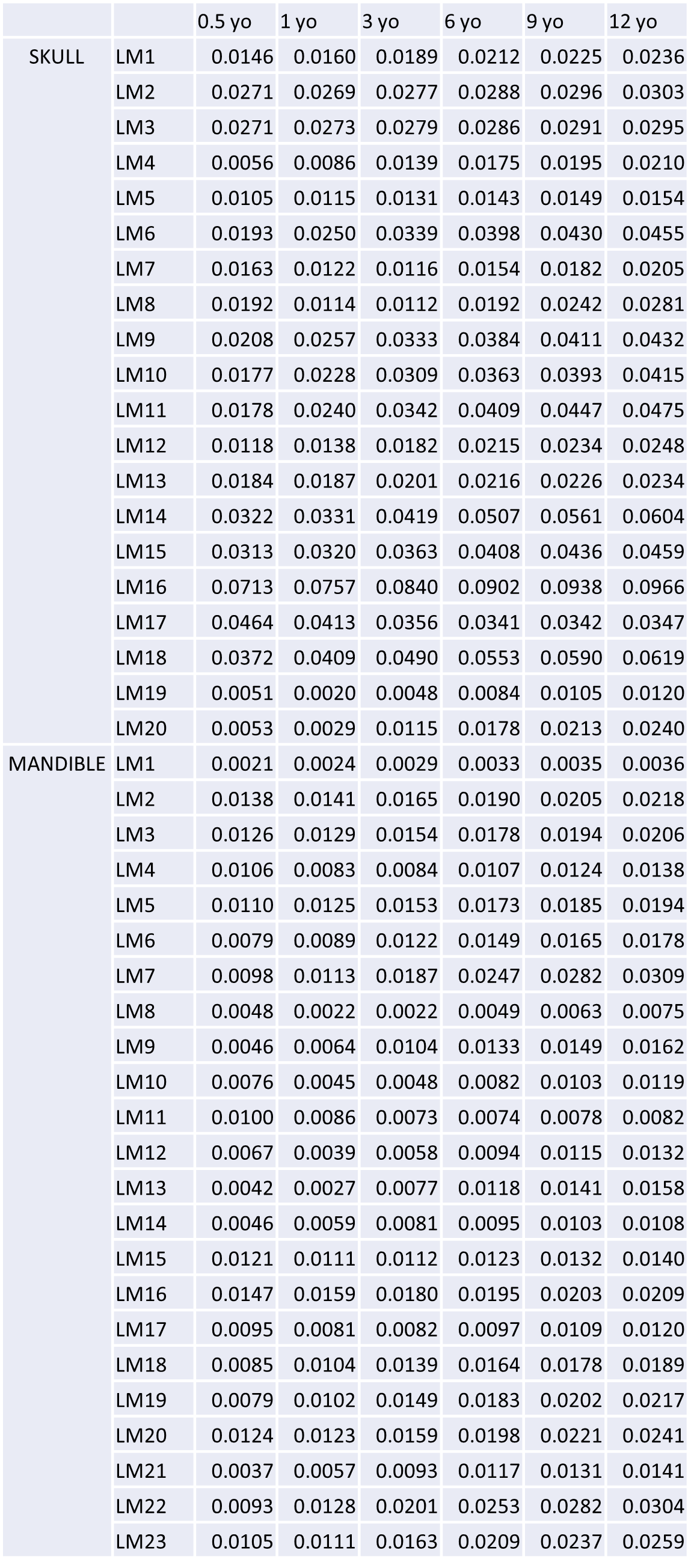

Supplement: Supplementary file 4 — Supplementary Material 4 [file 13023_2023_2664_MOESM4_ESM.png]

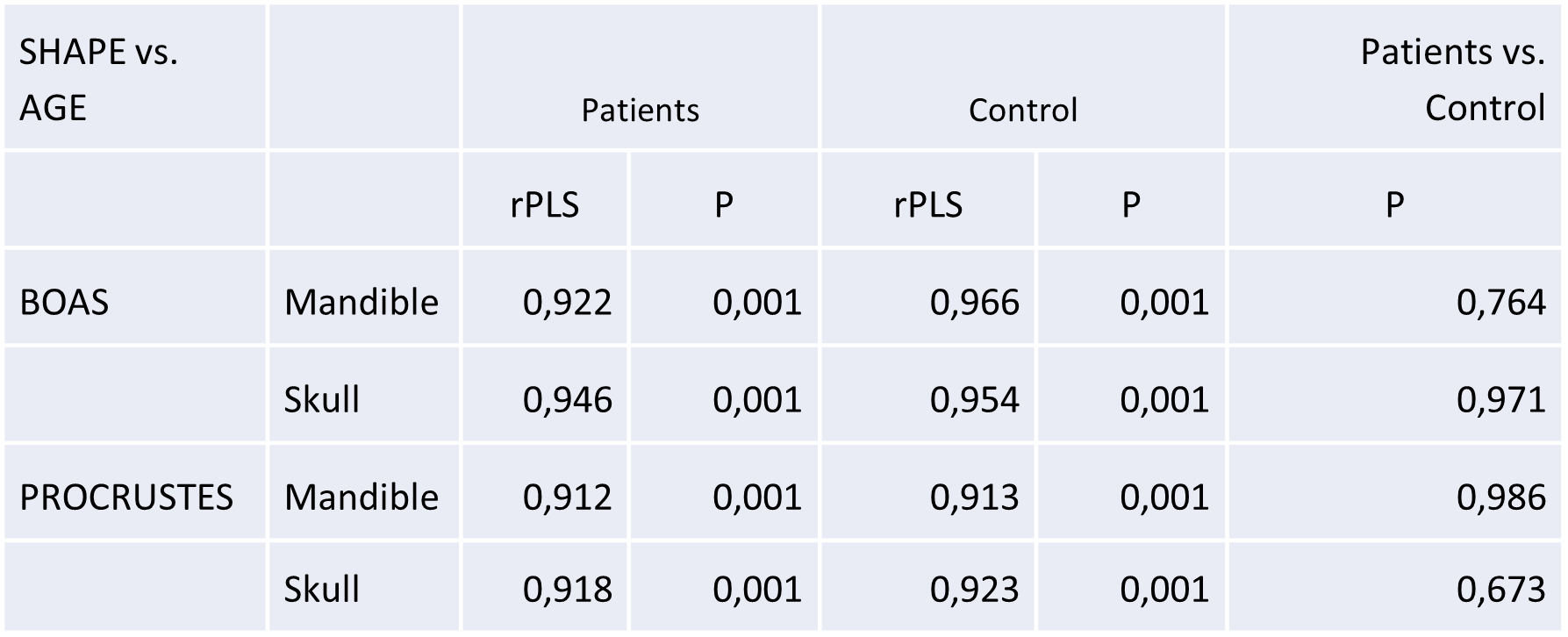

Supplement: Supplementary file 5 — Supplementary Material 5 [file 13023_2023_2664_MOESM5_ESM.png]

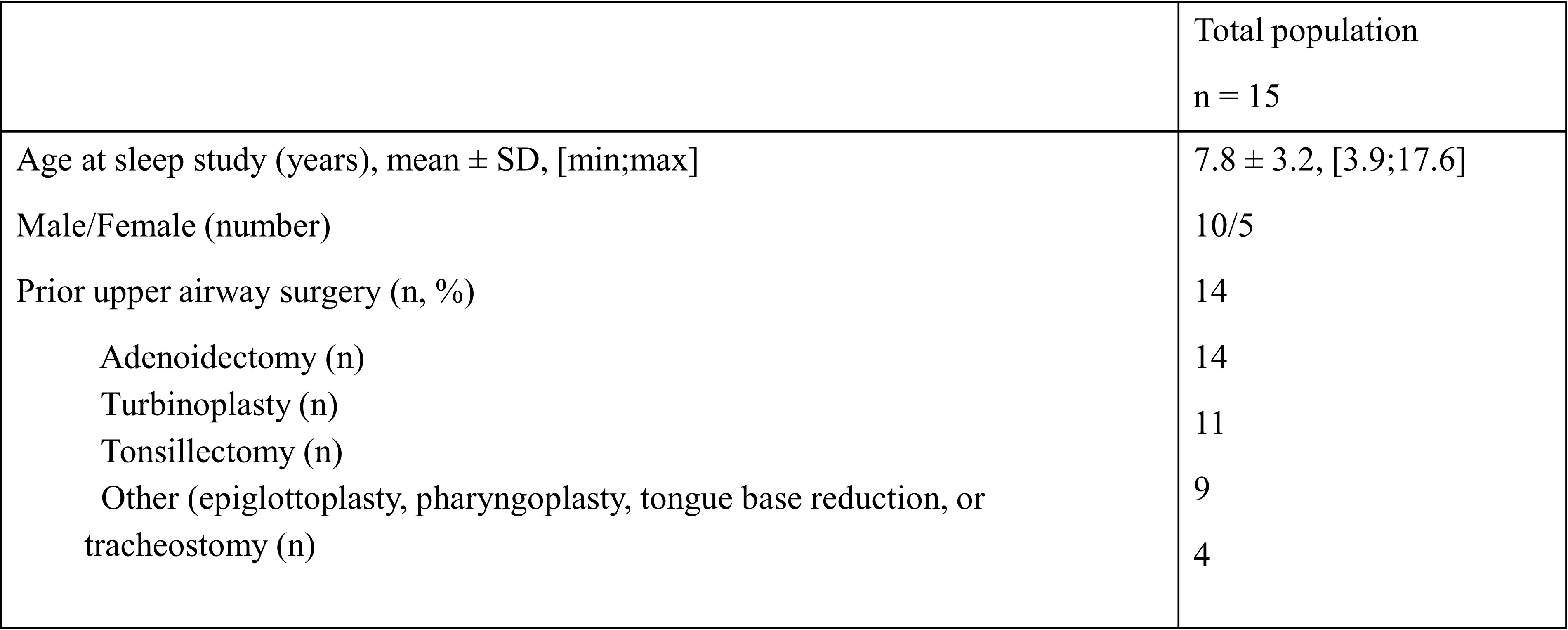

Supplement: Supplementary file 6 — Supplementary Material 6 [file 13023_2023_2664_MOESM6_ESM.jpg]

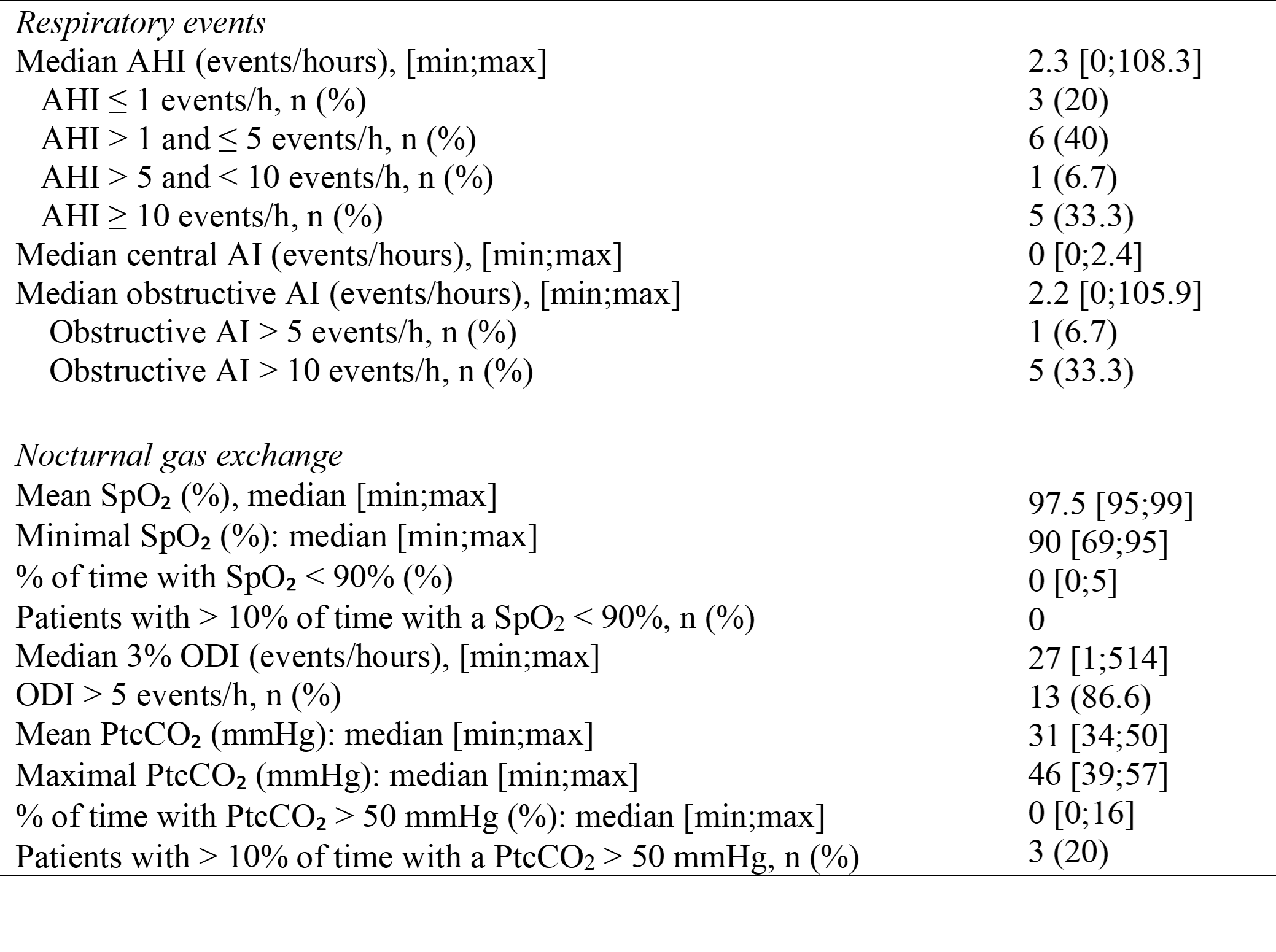

Supplement: Supplementary file 7 — Supplementary Material 7 [file 13023_2023_2664_MOESM7_ESM.png]

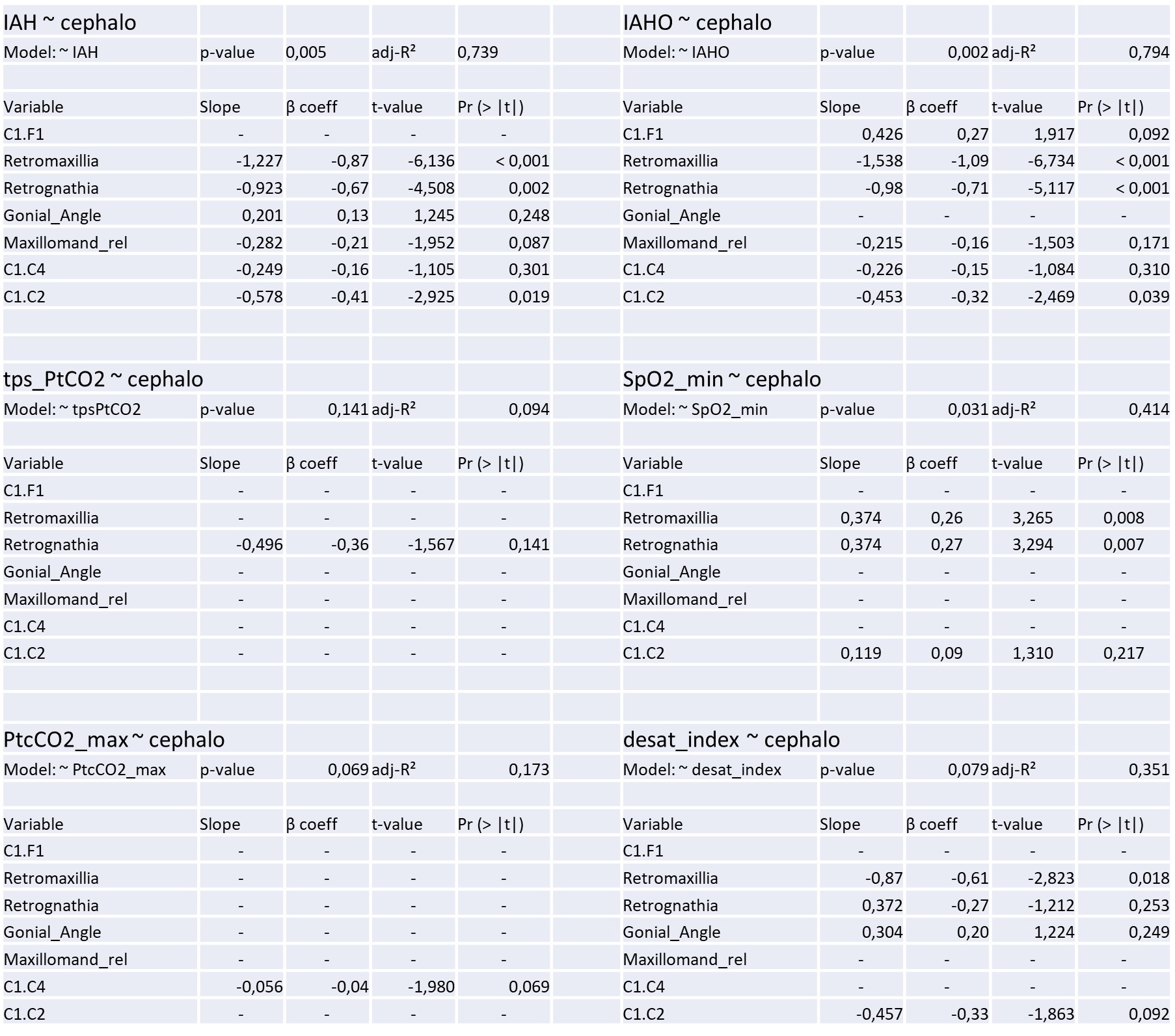

Supplement: Supplementary file 8 — Supplementary Material 8 [file 13023_2023_2664_MOESM8_ESM.jpg]

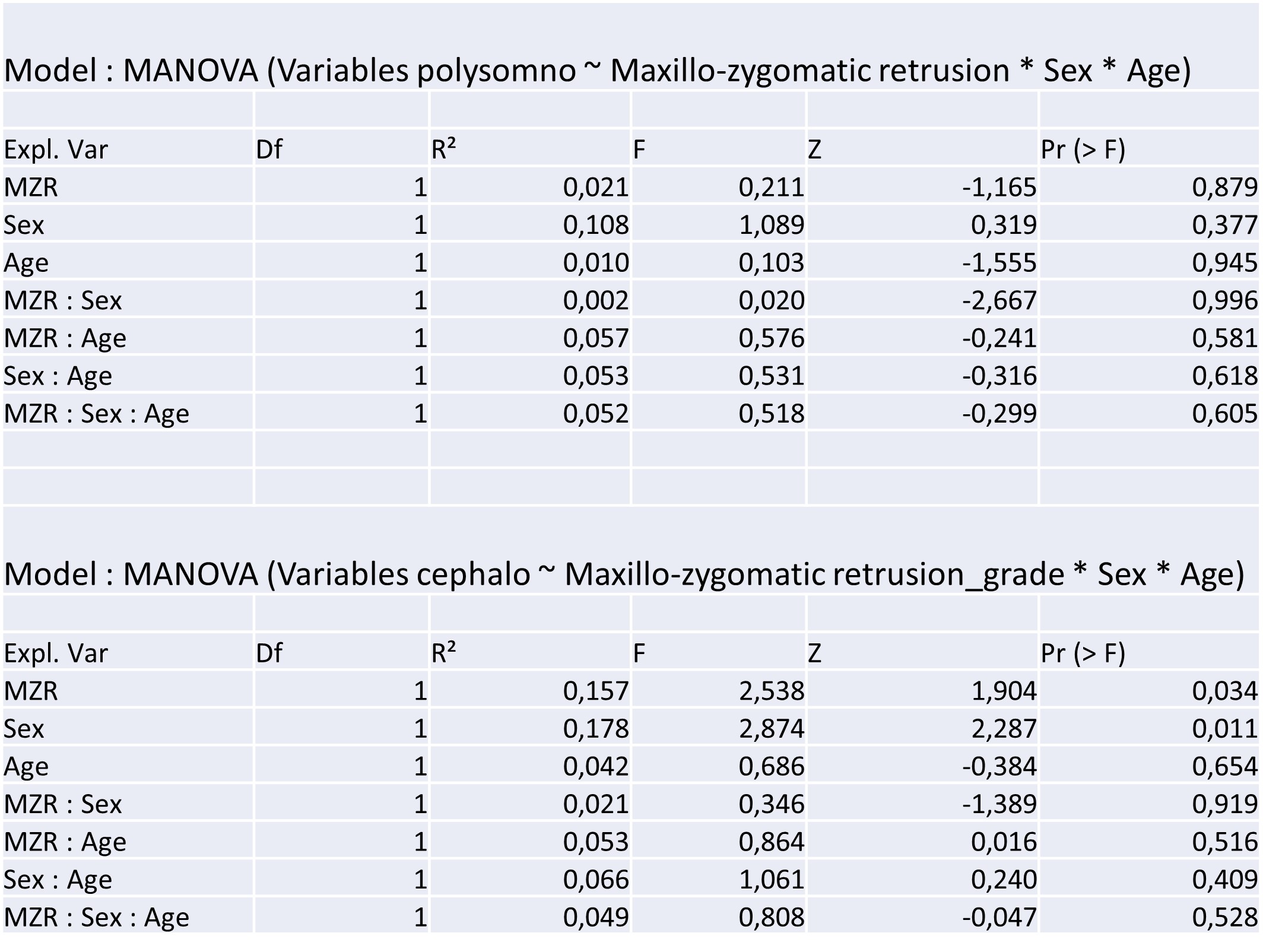

Supplement: Supplementary file 9 — Supplementary Material 9 [file 13023_2023_2664_MOESM9_ESM.jpg]

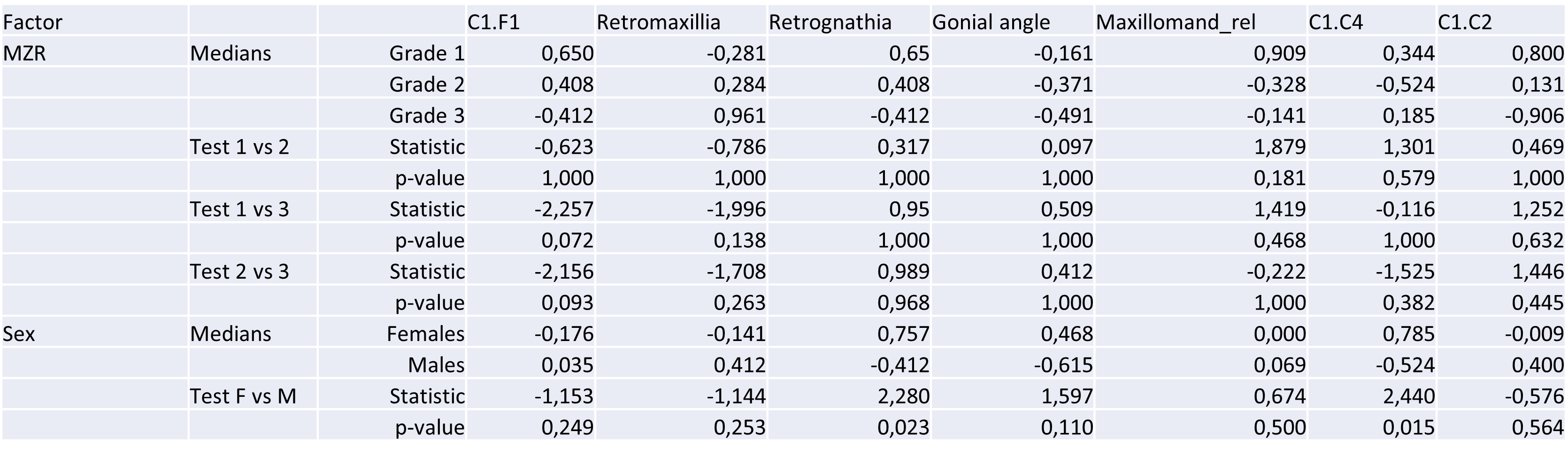

Supplement: Supplementary file 10 — Supplementary Material 10 [file 13023_2023_2664_MOESM10_ESM.png]
